# Theoretical Study on Structural Stability and Elastic Properties of Fe_25_Cr_25_Ni_25_Ti_x_Al_(25-x)_ Multi-Principal Element Alloys

**DOI:** 10.3390/ma14041040

**Published:** 2021-02-22

**Authors:** Li Liu, Ramesh Paudel, Yong Liu, Jing-Chuan Zhu

**Affiliations:** 1 Department of Materials Science and Engineering, Harbin Institute of Technology, Harbin 150001, China; lyonghit@hit.edu.cn; 2 Nepal Academy of Science and Technology (NAST), Khumaltar, Lalitpur 44700, Nepal; r.paudel@hit.edu.cn; 3 National Key Laboratory of Science and Technology on Advanced Composites in Special Environments, Harbin Institute of Technology, Harbin 150080, China

**Keywords:** Fe_25_Cr_25_Ni_25_Ti_x_Al_(25-x)_ alloys, atomic composition or configuration, microstructure, target properties

## Abstract

Material genetic engineering studies the relationship between the composition, microstructure, and properties of materials. By adjusting the atomic composition, structure, or configuration of the material and combining different processes, new materials with target properties obtained. In this paper, the design, and properties of the ordered phases in Fe_25_Cr_25_Ni_25_Ti_x_Al_(25-x)_ (subscript represents the atomic percentage) multi-principal element alloys are studied. By adjusting the percentages of Ti and Al atoms, the effect of the atomic percentage content on ordered phases’ structural stability in multi-principal element alloys are studied. Thermodynamic analysis predicted the composition phase and percentage of the alloy. Formation heat, binding energy, and elastic constants confirmed the structural stability and provide a theoretical basis for designing alloys with target properties. The results showed that the disordered BCC A2 phase and the ordered BCC B2 phase are the ductile phases, while the Laves phase is brittle. The research method in this paper is used to design multi-principal element alloys or other various complex materials that meet the target performance.

## 1. Introduction 

Multi-principal element alloys, also known as high-entropy alloys (HEAs), have been proven to have superior properties such as ultra-high strength, high hardness, high-temperature oxidation resistance, wear resistance, corrosion resistance, and thermal stability, in materials science [1,2,3,4,5,6,7] and have received considerable attention the engineering field. Compared with conventional alloys, multi-principal element alloys are alloys composed of five or more elements in equimolar ratios. The concentration of each element is between 5% and 35%, and no element content exceeds 50% [8,9]. Therefore, the main element alloy is characterized by the synergy of various elements. The design of multi-principal element alloys represents a new strategy for developing unique engineered materials with targeted properties that cannot be achieved with traditional alloy designs that rely on one element and then add other elements to improve the properties of the alloy.

At present, studies on multi-principal element alloys show that the main factor affecting the entropy value is still the structure of the alloy. Secondly, due to the different atom types and sizes of the multi-principal element alloys, a severe lattice distortion is caused, and the shear modulus between the constituent atoms does not match, which may contribute to hardening. The structural classification of a multi-principal element alloys is classified into one or more microstructures composed of disordered solid solutions. Microstructures having one or more ordered phases (or intermetallic compounds) and a disordered solid solution A microstructure consisting of a mixture of intermetallic phases. The most common constituent structures, at present, are the disordered FCC phase and the BCC phase, followed by the ordered BCC B2 phase and the hexagonal Laves phase.

For the study of multi-principal element alloys, the empirical method is to calculate the formation of the disordered solid solution by the Hume-Rothery rule. The factors considered are the atomic size factor, crystal structure, electronegativity, electron concentration, and valence. The conditions for thermodynamic formation are mixed enthalpy, mixing entropy, and melting temperature [10]. The Gibbs free energy is obtained by establishing the equation by the number and concentration of alloying elements without considering the Gibbs free energy of the ordered phase or intermetallic compound. Another method can be used to form entropy and an intermetallic phase to create a crucible. The modeling can distinguish whether the alloy is a single-phase disordered solid solution or contains an ordered phase (intermetallic compound). The third method is the phase diagram calculation, obtaining the phase diagram of the multi-principal element alloys, analyzing the phase structure, and the phase volume fraction. The mechanical properties of the multi-principal element alloys depend, to a large extent, on the composition and microstructure of the alloy. In the case of fixed composition and phase content, the size, shape, and distribution of the phase can also be adjusted to improve the properties of the alloy. Second, factors affecting mechanical properties include defects such as vacancies, dislocations, and microscopic defects such as grain boundaries, as well as the porosity, segregation, cracks, and residual stress. For the study of the hardness of Al_x_CoCrCuFeNi multi-principal element alloys, the structure changed from single-phase fcc to BCC + FCC and then to single-phase BCC structure, and its hardness increased with the increase of Al content [11]. It has also shown that in the Al_x_CoCrCuFeNi multi-principal element alloys, since it contains a large electronegativity difference element such as Al/Ni, increasing the Al content leads to chemical order, which acts as a solid solution strengthening and precipitation strengthening, so the hardness increases [12,13,14]. In the study of CrCoFeNiMn multi-principal element alloys, the grain size and strain rate are the main factors affecting the tensile properties. The method of cooking material design has become an effective way to design new materials [15,16].

Based on the research ideas of material genetic engineering, the chemical composition, phase composition, structure, and properties of Fe_25_Cr_25_Ni_25_TixAl_(25−x)_ (x = 0; 6.25; 12.5; 18.75; 25) (Subscript represents atomic percentage) multi-principal element alloys were studied through the thermodynamic phase diagram calculation, first-principles calculation and experimental verification methods. The relationship between the purposes is to design a new multi-principal element alloys, hoping to improve the ductility of the alloy. The thermodynamic phase diagram predicted the phase composition and content of the alloy and the main constituent elements of the phase. Then the stability and elastic properties of the phase structure were predicted by first-principles calculation. Finally, the reliability of the estimate was verified by experiments.

## 2. Calculation Methods

### 2.1. Thermodynamic Methods

For many types of alloys, the advantage of being able to calculate their thermo physical properties through JMatPro software is obvious. We can easily obtain information about the alloy and calculate where the alloy cannot be measured. Because of the lack of obvious and reliable measurement of solidification performance for many alloys, this is a precious advantage. The change of alloy phase content with alloy composition and allowable specification range can be obtained quickly and self-consistently through calculation. Beside, many calculation paths are accompanied by detailed information; otherwise, it is impossible or complicated to determine experimentally. For example, in FeCrNiTi_x_Al_(1-x)_ multi-principal alloy. Due to the lack of data on thermo physics and physics, the properties of similar alloys can be given through calculations. Some alloys are very sensitive to small composition changes, while other alloys (for example, solid solution alloys) may not be sensitive. The composition specification may also be because the multi-principal alloy is very wide, and many researchers have taken advantage of this alloy.

The thermodynamic calculation software JMatPro used in this article is Public Release Version 7.0.0 provided by Sente Software Ltd. Surrey Technical Center [17,18,19]. Use the nickel-based alloy module of the software to calculate Fe_25_Cr_25_Ni_25_Al_25_, Fe_25_Cr_25_Ni_25_Ti_6.25_Al_18.75_, Fe_25_Cr_25_Ni_25_Ti_12.5_Al_12.5_, Fe_25_Cr_25_Ni_25_Ti_18.75_Al_6.25_, Fe_25_Cr_25_Ni_25_Ti_25_, and Fe_25_Cr_25_Ni_25_Ti_x_Al_(25-x)_ (x = 0; 6.25; 12.5; 18.75; 25) (subscript represents atomic percentage). General physical properties of multi-principal element alloys, alloy phases and cooling curves, the setting temperature range are 0–1400 °C. The phase composition and content of the alloy during solidification were studied. 

### 2.2. Density Functional Theory

The plane wave ultra-soft pseudopotential method based on density functional theory has been widely applied to the structural design and performance prediction of materials. Based on this method, the first principle modeling of the constituent phases in the alloy is carried out according to the calculation results of the phase diagram. Using the CASTEP module in Materials Studio 8.0 [20], select the utilized density functional PBE in generalized gradient approximation (GGA) [21], select 350 eV for the energy cutoff value, and select k point for 4 × 4 × 4 in the first Brillouin region of the reciprocal space. The volume and atomic position of the supercell structure optimized until the maximum force, maximum displacement, maximum stress, and energy change of each atom are below 0.01 eV/Å, 5.0 × 10^−4^ Å, 0.02 GPa, and 5.0 × 10^−6^ eV/atom, respectively. The stability and elastic properties of the alloy structure, such as second-order elastic constant, bulk modulus, shear modulus, Young’s modulus, and Poisson’s ratio were comprehensively calculated. 

## 3. Results and Discussion

### 3.1. Thermodynamic Analysis

To study the types and relative contents of various constituent phases of Fe_25_Cr_25_Ni_25_Ti_x_Al_(25−x)_ (x = 0; 6.25; 12.5; 18.75; 25) (Subscript represents atomic percentage) multi-principal element alloys at different temperatures, and to determine the composition of each equilibrium phase, thermodynamic analysis The software JMat-Pro performs phase diagram calculation on it. To combine the thermodynamic analysis with the order of the structure in the multi-principal element alloys, try to combine the thermodynamics with the first-principles calculations to explore a new multi-principal alloy that meets the target performance.

**Figure 1:**
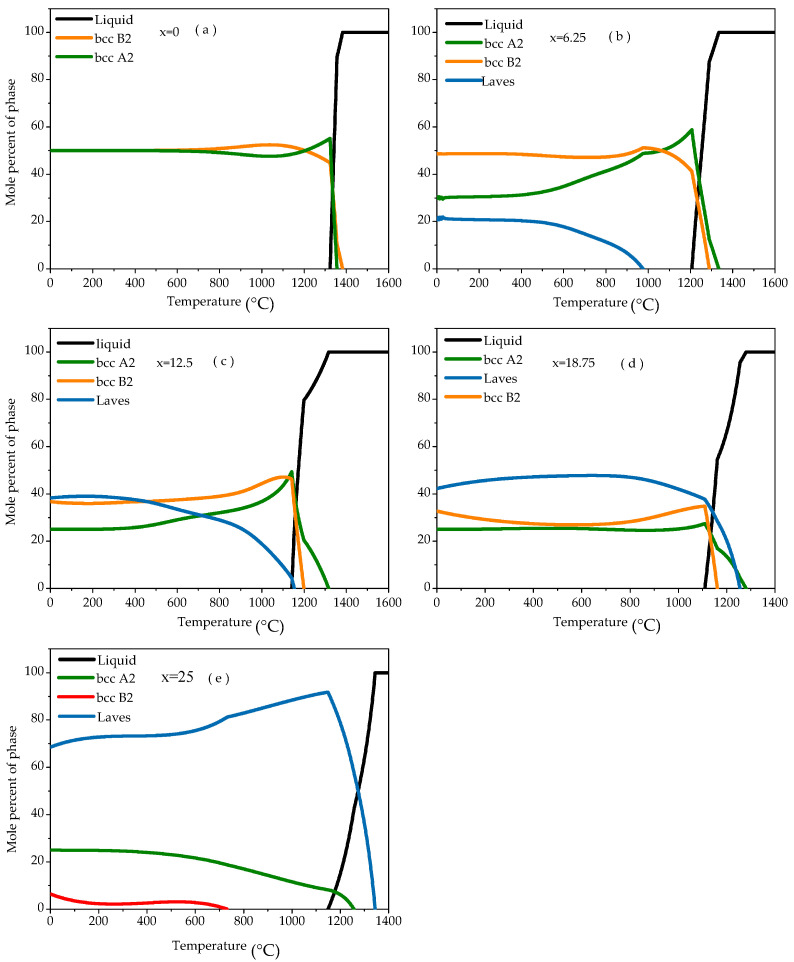
Relationship of phase content and temperature for Fe_25_Cr_25_Ni_25_Ti_x_Al_(25−x)_ multi-principle element alloys. (**a**) x = 0; (**b**) x = 6.25; (**c**) x = 12.5; (**d**) x = 18.75; (**e**) x = 25.

Among the constituent elements of the Fe_25_Cr_25_Ni_25_Ti_x_Al_(25−x)_ (subscript represents atomic percentage) system multi-principal element alloys, the Fe, Ni, and Ti elements are the main constituent elements of the conventional metal-based superalloy. The Ni, Ti, and Cr elements contribute to the high-temperature properties of traditional alloys. Due to the large atomic radius of the Al element, it has a great influence on the crystal structure of the alloy system. The crystal structure of the alloy can be adjusted by changing the content of the Al element to achieve the purpose of improving the properties of the alloy. In the Fe_25_Cr_25_Ni_25_Ti_x_Al_(25−x)_ system multi-principal element alloys, it is desirable to adjust the content of the Ti and Al elements to improve the toughness. Figure 1 shows the thermodynamic calculation results for the alloy. When x = 0, the Fe_25_Cr_25_Ni_25_Al_25_ alloy phase diagram shows that the disordered BCC A2 phase and the ordered BCC B2 phase content are 50%, respectively (Figure 1a). As the Ti content increases (x = 6.25), the Al content reduces, the content of the ordered BCC B2 phase and the disordered BCC A2 phase increased simultaneously, and the Laves phase decreased (Figure 1b). When x = 12.5, the content of the disordered BCC A2 phase changes little, the content of the ordered BCC B2 phase continues to increase, and the Laves phase content continues to decrease (Figure 1c). As the Ti content continued to increase (x = 18.75), the Al content continued to reduce, the content of the disordered BCC A2 phase and the ordered BCC B2 phase increases, and the Laves phase content decreases, less than 50% (Figure 1d). When x = 25, the phase diagram of the Fe_25_Cr_25_Ni_25_Ti_25_ alloy shows that the alloy consists of a disordered BCC A2 phase, an ordered BCC B2 phase, and a Laves phase, a small BCC B2 phase content, and a Laves phase content greater than 75% (Figure 1e). Further analysis showed that with the increase of Ti content and the decrease of Al content, the content of Al phase and B2 phase decreased gradually, the content of A2 phase decreased rapidly, and the content of Laves phase increased gradually. Table 1 shows the mole percent of each phase of the Fe_25_Cr_25_Ni_25_Ti_x_Al_(25-x)_ alloy at 800 °C. It can be seen from the data in the table that as the Al content in the alloy increases, the contents of the BCC A2 phase and the BCC B2 phase gradually increase. The Laves phase content is gradually reduced.

### 3.2. Solidification Physical Properties

**Figure 2:**
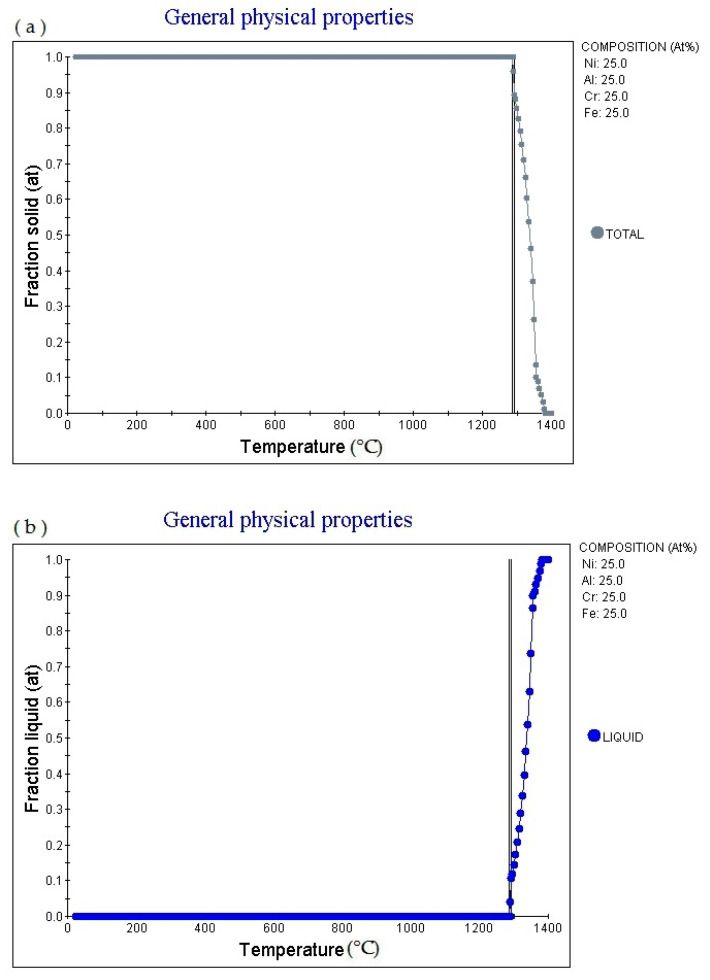

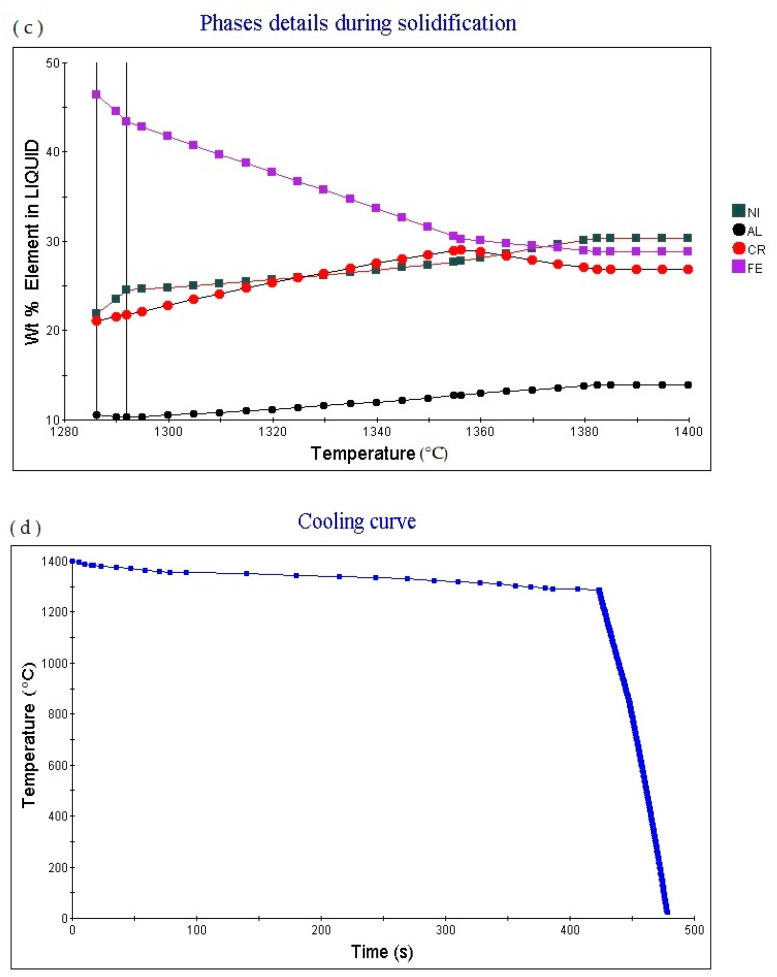
Solidification physical properties. Physical properties for Fe_25_Cr_25_Ni_25_Al_25_ multi-principle element alloys. (**a**) general physical properties (fraction solid–temperature); (**b**) general physical properties (fraction liquid–temperature); (**c**) wt.% element in liquid–temperature; (**d**) cooling curve (temperature–time).

Figure 2a is the Fraction solid (at)-Temperature (°C) curve of Fe_25_Cr_25_Ni_25_Al_25_ alloy (subscript as atomic percentage). The figure shows that as the temperature increases, the alloy gradually melts, and the melting point is 1288.53 °C: 1.0. Then as the temperature increases, the solid phase volume fraction gradually decreases. When the temperature increases to 1382.36 °C, Fraction solid (at): 0.0. Figure 2b is the fraction liquid (at)–temperature (°C) curve of Fe_25_Cr_25_Ni_25_Ti_18.75_Al_6.25_ alloy (subscript is atomic percentage). The figure shows that as the temperature increases, the volume fraction of the liquid phase in the alloy changes accordingly. The multi-principal element alloy has a fixed melting temperature, the melting temperature is 1284.85 °C, currently fraction liquid (at): 0.0. It absorbs heat during the melting process. When the temperature rises to 1356.1 °C, fraction Liquid (at): 0.9. Then as the temperature increases, the alloy gradually melts. When the temperature increases to 1382.36 °C, fraction liquid (at): 1.0. Figure 2c shows the element in liquid (wt.%)—temperature curve of the alloy. As the temperature increases, the percentage content of various elements in the alloy liquid phase will change. Figure 2d shows the cooling curve of the alloy. The cooling rate obtained for the cooling curve is 1.0 °C/S. When the cooling time is 423.31 s and the temperature is 1288.53 °C, the cooling rate can be adjusted to obtain cooling curves with different cooling rates.

**Figure 3:**
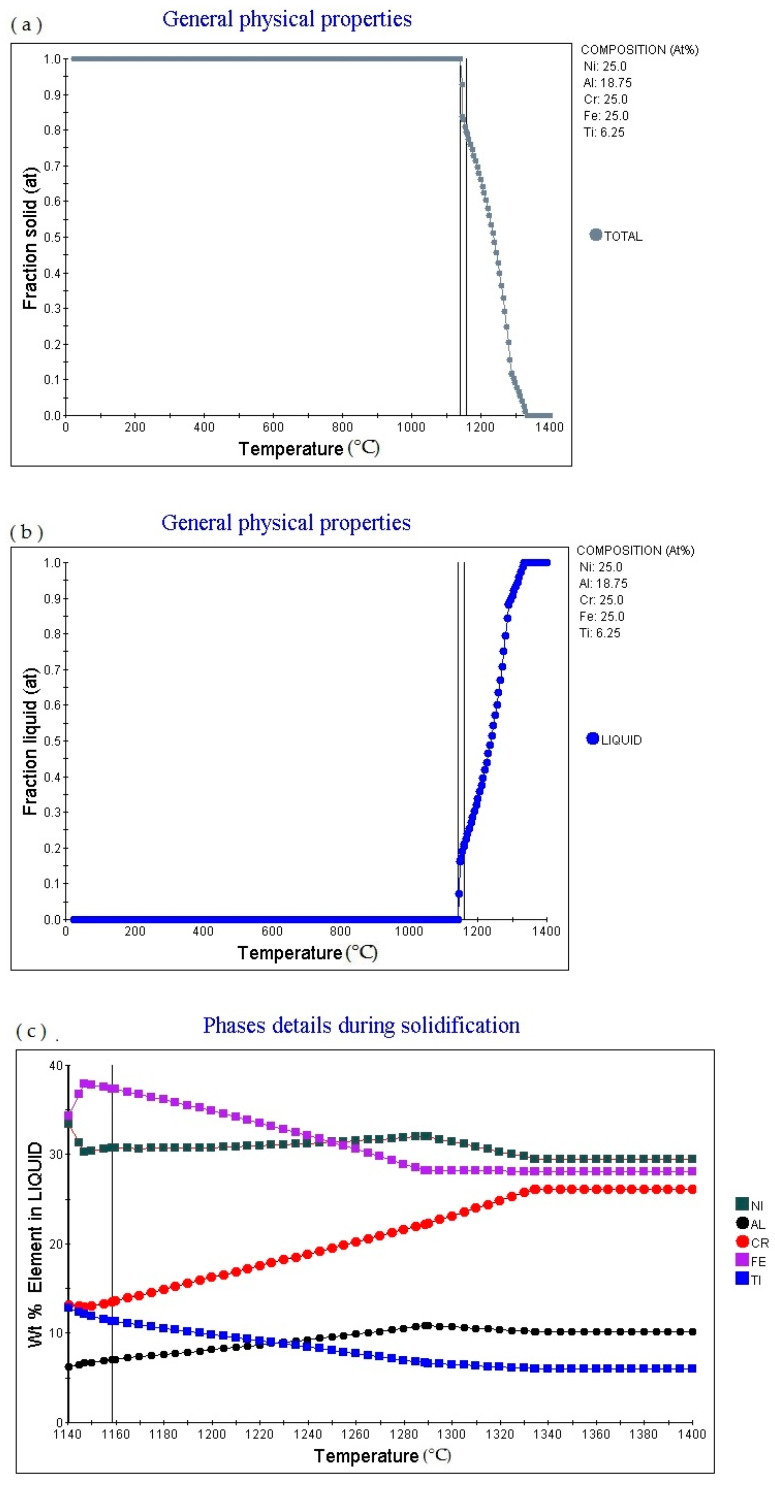

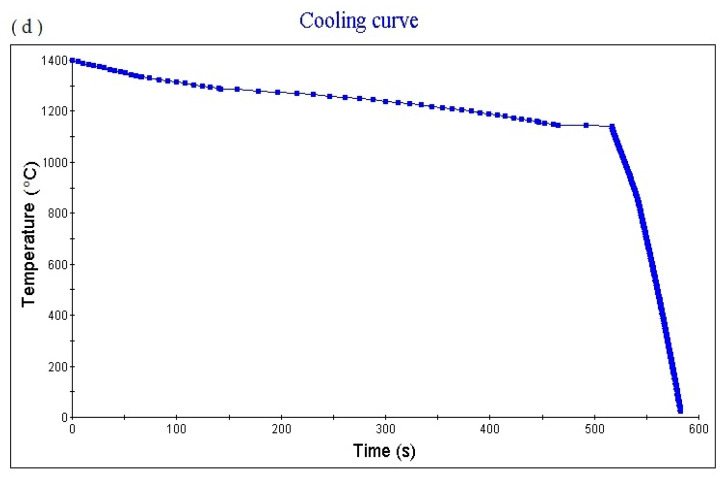
Solidification physical properties for Fe_25_Cr_25_Ni_25_Ti_6.25_Al_18.75_ multi-principle element alloys. (**a**) general physical properties (fraction solid–temperature); (**b**) general physical properties (fraction liquid–temperature); (**c**) wt.% element in liquid–temperature; (**d**) cooling curve (temperature–time).

Figure 3a is the fraction solid (at)–temperature (°C) curve of Fe_25_Cr_25_Ni_25_Ti_6.25_Al_18.75_ alloy (subscript is atomic percentage). The figure shows that as the temperature rises, the alloy gradually melts, and the melting point is 1140.41 °C. Fraction solid (at): 1.0. Then as the temperature increases, the solid phase volume fraction gradually decreases. When the temperature increases to 1334.85 °C, fraction solid (at): 0.0. Figure 3b is the fraction liquid (at)–temperature (°C) curve of Fe_25_Cr_25_Ni_25_Ti_18.75_Al_6.25_ alloy (subscript is atomic percentage). The figure shows that as the temperature increases, the volume fraction of the liquid phase in the alloy changes accordingly. The multi-principal element alloy has a fixed melting temperature, which is 1140.41°C, currently fraction liquid (at): 0.0. It absorbs heat during the melting process. When the temperature rises to 1146.89 °C, fraction liquid (at): 0.16. When the temperature rises to 1289.85 °C, fraction liquid (at): 0.88. Then as the temperature rises, the alloy gradually melts. When the temperature rises to 1334.85 °C, fraction liquid (at): 1.0. As the temperature rises, fraction liquid (at) remains unchanged. Figure 3c shows the element in liquid (wt.%)—temperature curve of the alloy. As the temperature increases, the percentage content of various elements in the alloy liquid phase will change. Figure 3d shows the cooling curve of the alloy. The cooling rate obtained for the cooling curve is 1.0 °C/s. when the cooling time is 516.5 s and the temperature is 1140.41 °C, the cooling rate can be adjusted to obtain cooling curves with different cooling rates.

**Figure 4:**
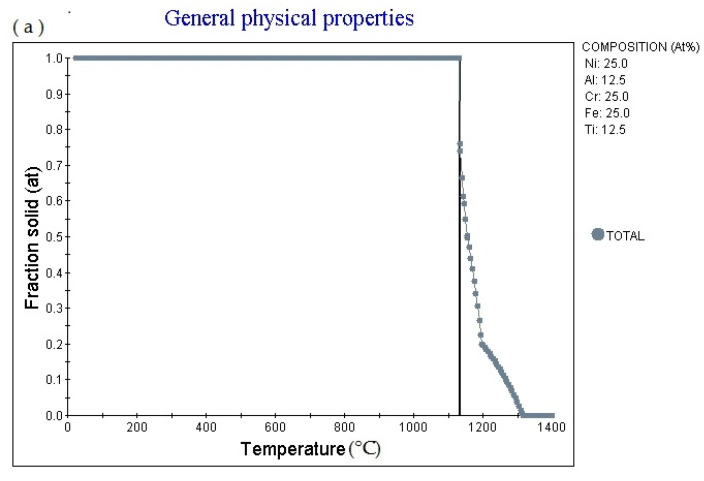

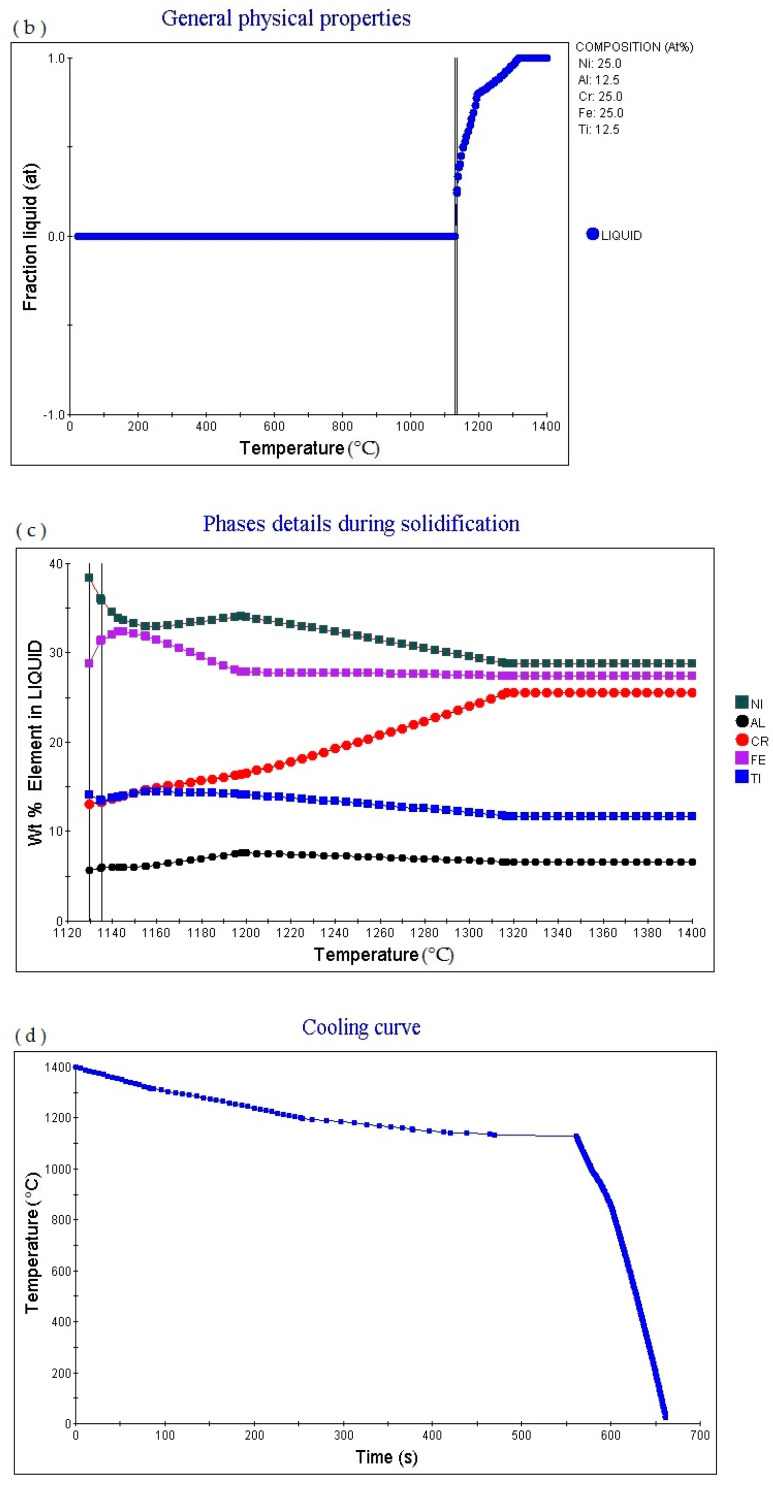
Solidification physical properties. Physical properties for Fe_25_Cr_25_Ni_25_Ti_12.5_Al_12.5_ multi-principle element alloys. (**a**) general physical properties (fraction solid–temperature); (**b**) general physical properties (fraction liquid–temperature); (**c**) wt.% element in liquid–temperature; (**d**) cooling curve (temperature–time).

Figure 4a is the Fraction solid (at)-Temperature (°C) curve of Fe_25_Cr_25_Ni_25_Ti_12.5_Al_12.5_ alloy (subscript is atomic percentage). The figure shows that as the temperature rises, the alloy gradually melts, and the melting point is 1129.91 °C, currently Fraction solid (at): 1.0. Then as the temperature increases, the solid phase volume fraction gradually decreases. When the temperature increases to 1316.71 °C, Fraction solid (at):0.0. Figure 4b is the fraction liquid (at)–temperature (°C) curve of Fe_25_Cr_25_Ni_25_Ti_12.5_Al_12.5_ alloy (subscript is atomic percentage). The figure shows that as the temperature increases, the volume fraction of the liquid phase in the alloy changes accordingly Variety. The multi-principal element alloys has a fixed melting temperature, the melting temperature is 1129.91 °C, at this time fraction liquid (at): 0.0. It absorbs heat during the melting process. When the temperature rises to 1199.85 °C, fraction liquid (at): 0.8. Then as the temperature rises, the alloy gradually melts. When the temperature rises to 1314.85 °C, fraction liquid (at): 1.0. As the temperature rises, the fraction liquid (at) remains unchanged. Figure 4c shows the element in liquid (wt.%)—temperature curve of the alloy. As the temperature increases, the percentage content of various elements in the alloy liquid phase will change. Figure 4d shows the cooling curve of the alloy. The cooling rate obtained for the cooling curve is 1.0 °C/s. When the cooling time is 561.03 s, the temperature is 1129.91 °C. The cooling rate can be adjusted to obtain cooling curves with different cooling rates. 

**Figure 5:**
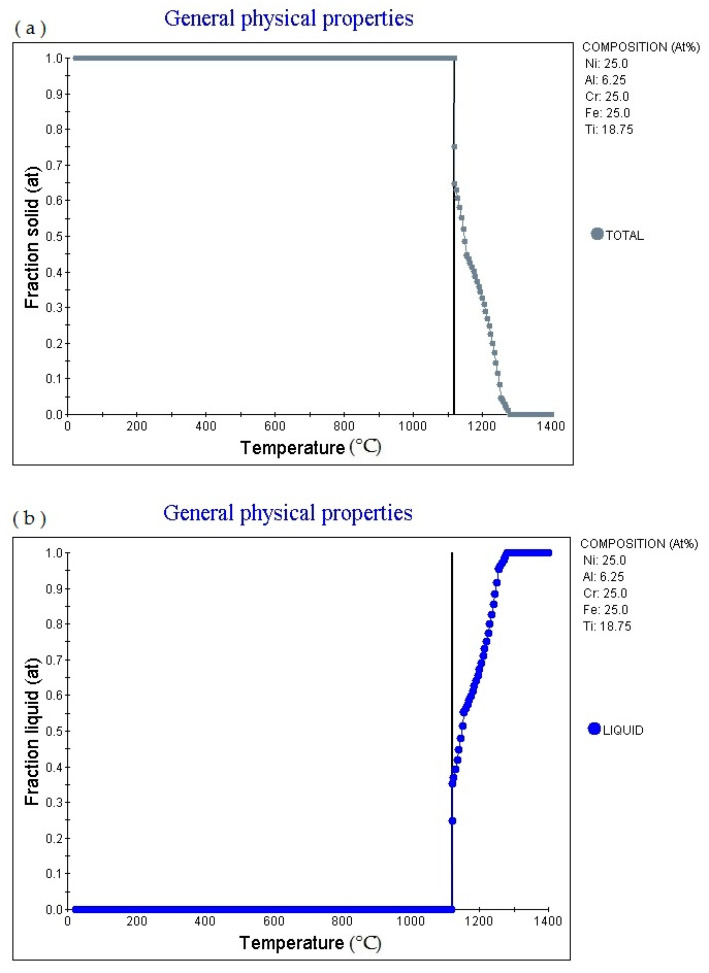

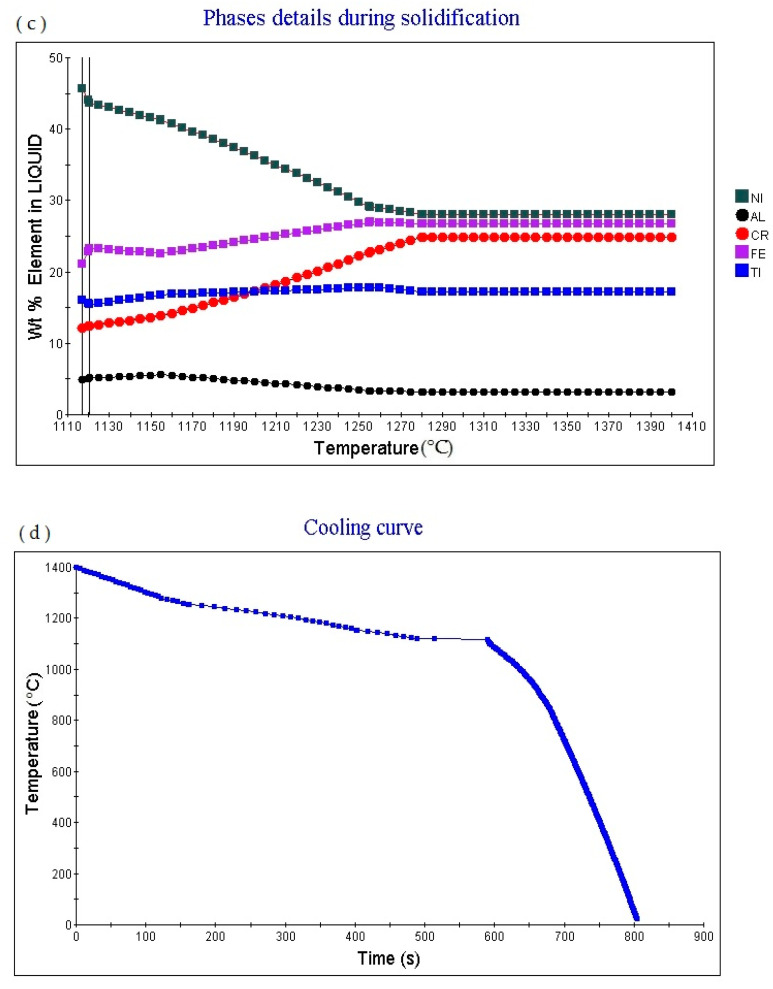
Solidification physical properties Physical properties for Fe_25_Cr_25_Ni_25_Ti_18.75_Al_6.25_ multi-principle element alloys. (**a**) general physical properties (fraction solid–temperature); (**b**) general physical properties (fraction liquid–temperature); (**c**) wt.% element in liquid–temperature; (**d**) cooling curve (temperature–time).

Figure 5a is the Fraction solid (at)-temperature (°C) curve of Fe_25_Cr_25_Ni_25_Ti_18.75_Al_6.25_ alloy (subscript is atomic percentage). The figure shows that as the temperature rises, the alloy gradually melts, with a melting point of 1117.17 °C, currently Fraction solid (at): 1.0. Then as the temperature increases, the solid phase volume fraction gradually decreases. When the temperature increases to 1280.37 °C, Fraction solid (at): 0.0. Figure 5b is the fraction liquid (at)–temperature (°C) curve of Fe_25_Cr_25_Ni_25_Ti_18.75_Al_6.25_ (subscript is atomic percentage), which shows that as the temperature increases, the volume fraction of the liquid phase in the alloy changes accordingly. The multi-principal element alloy has a fixed melting temperature, which is 1117.17 °C, at this time fraction liquid (at): 0.0. As the temperature rises, the alloy gradually melts. When the temperature rises to 1280.37 °C, fraction liquid (at): 1.0. As the temperature rises, fraction liquid (at) remains unchanged. Figure 5c shows the element in liquid (wt.%)—temperature curve of the alloy. As the temperature increases, the percentage content of various elements in the alloy liquid phase will change. Figure 5d shows the cooling curve of the alloy. The cooling rate of the obtained cooling curve is 1.0 °C/S. When the cooling time is 589.38 s, the temperature is 1117.92 °C. The cooling rate can be adjusted to obtain cooling curves with different cooling rates.

**Figure 6:**
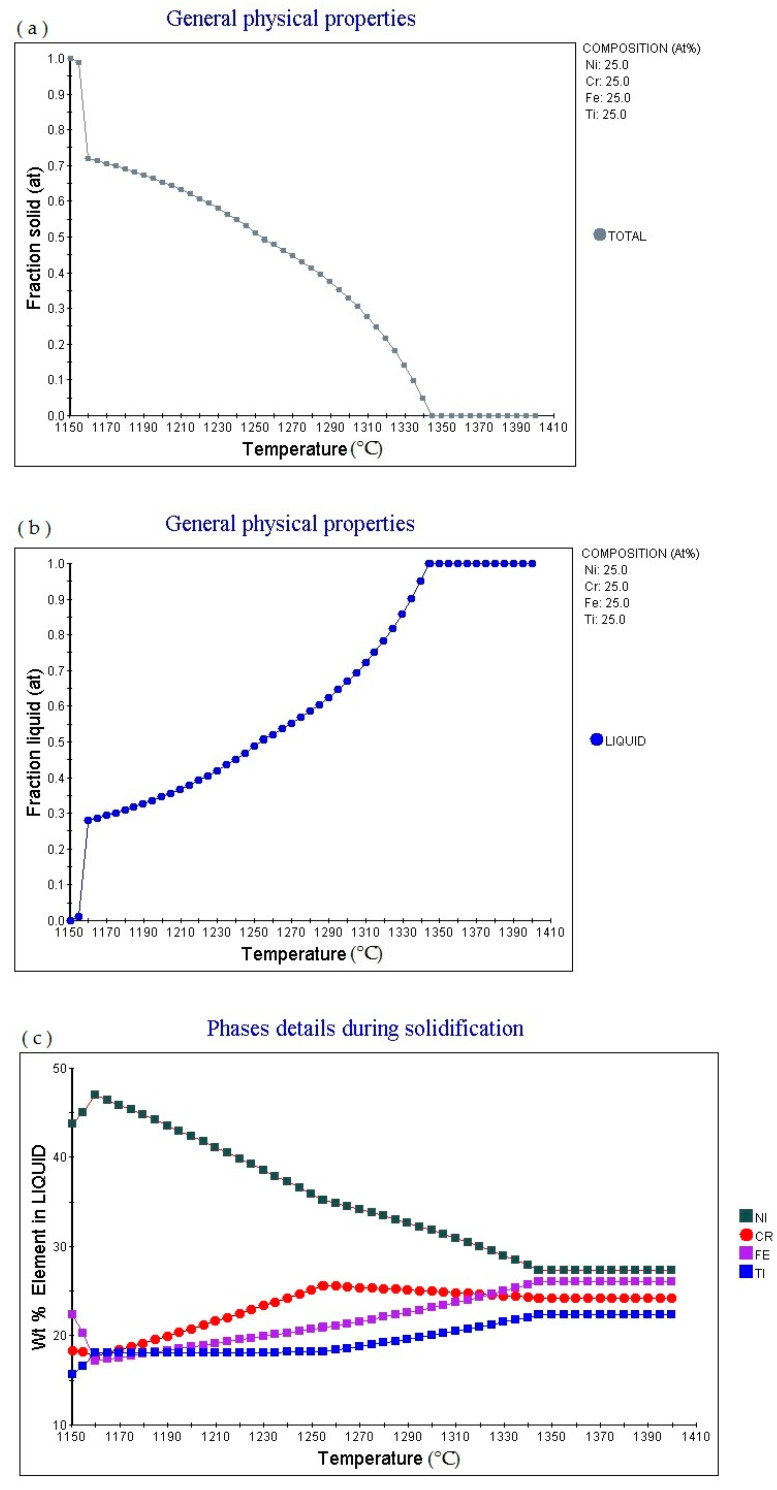

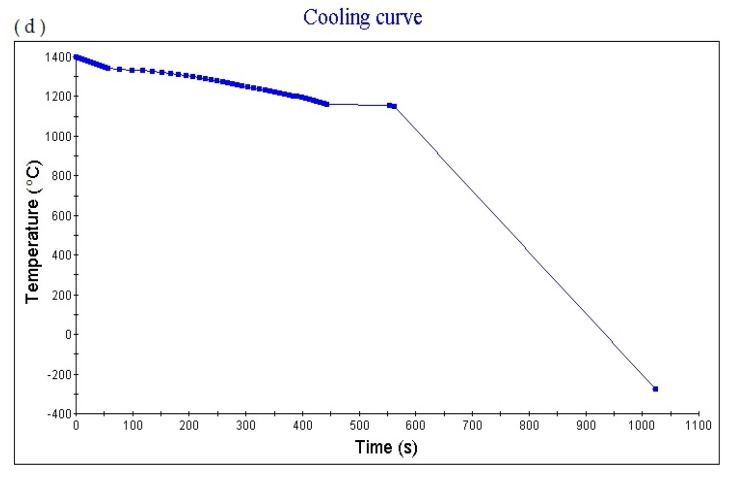
Solidification physical properties Physical properties for Fe_25_Cr_25_Ni_25_Ti_25_ multi-principle element alloys. (**a**) General physical properties (fraction solid–temperature); (**b**) general physical properties (fraction liquid–temperature); (**c**) wt.% element in liquid–temperature; (**d**) cooling curve (temperature–time).

Figure 6a is the Fraction solid (at)-Temperature (°C) curve of Fe_25_Cr_25_Ni_25_Ti_25_ alloy (subscript is atomic percentage). The figure shows that as the temperature increases, the alloy gradually melts. When the temperature is 1150.53 °C, Fraction solid (at): 1.0. Then as the temperature increases, the solid phase volume fraction gradually decreases. When the temperature increases to 1159.85 °C, Fraction solid (at): 0.72; when the temperature increases to 1344.85 °C, Fraction solid (at): 0.0. Figure 6b is the fraction liquid (at)—temperature (°C) curve of Fe_25_Cr_25_Ni_25_Ti_25_ (subscript as atomic percentage). The figure shows that as the temperature increases, the volume fraction of the liquid phase in the alloy changes accordingly. The multi-principal element alloys has a fixed melting temperature, the melting temperature is 1159.85 °C, at this time fraction liquid (at): 0.0. As the temperature rises, the alloy gradually melts. When the temperature rises to 1159.85 °C, fraction liquid (at): 0.28; when the temperature rises to 1344.44 °C, fraction liquid (at): 1.0. As the temperature rises, fraction liquid (at) remains unchanged. Figure 6c shows the element in liquid (wt.%)-temperature curve of the alloy. As the temperature increases, the percentage content of various elements in the alloy liquid phase will change. Figure 6d shows the cooling curve of the alloy. The cooling rate of the obtained cooling curve is 1.0 °C/s. When the cooling time is 561.6 s and the temperature is 1150.53 °C, the cooling rate can be adjusted to obtain cooling curves with different cooling rates.

### 3.3. Enthalpy of Mixing ΔHmix, Entropy of Mixing ΔSmix and Gibbs Free Energy Calculation of Alloy

In thermodynamics, the entropy value represents the degree of chaos. The greater the degree of disorder in a system, the greater the entropy value, as shown in Formula (1) [22]:

(1) $$\Delta S_{mix}=-R\overset{n}{\underset{i}{\sum }}C_{i}\ln C_{i}$$

R: Represents the gas constant; $C_{i}$: Represents the percentage of member *i*.

In multi-principal alloys, the mixing entropy plays a significant role in the formation of a solid solution, and the mixing enthalpy factor cannot be ignored. The formula for mixing enthalpy is shown in Formulas (2) and (3) [23]:

(2) $$\Delta H_{\mathrm{mix}}=\overset{n}{\underset{i=1,i\neq j}{\sum }}\Omega_{ij}C_{i}C_{j}$$

(3) $$\Omega_{ij}=4\times \Delta {H\;}_{\mathrm{AB}\;}^{\mathrm{mix}}$$

$\;C_{i}$: The atomic percentage of the *i* component;$\;C_{j}$: The atomic percentage of the *j* component;$\;\Delta H_{\mathrm{AB}\;}^{\mathrm{mix}}$ binary system mixing enthalpy. 

**Figure 7:**
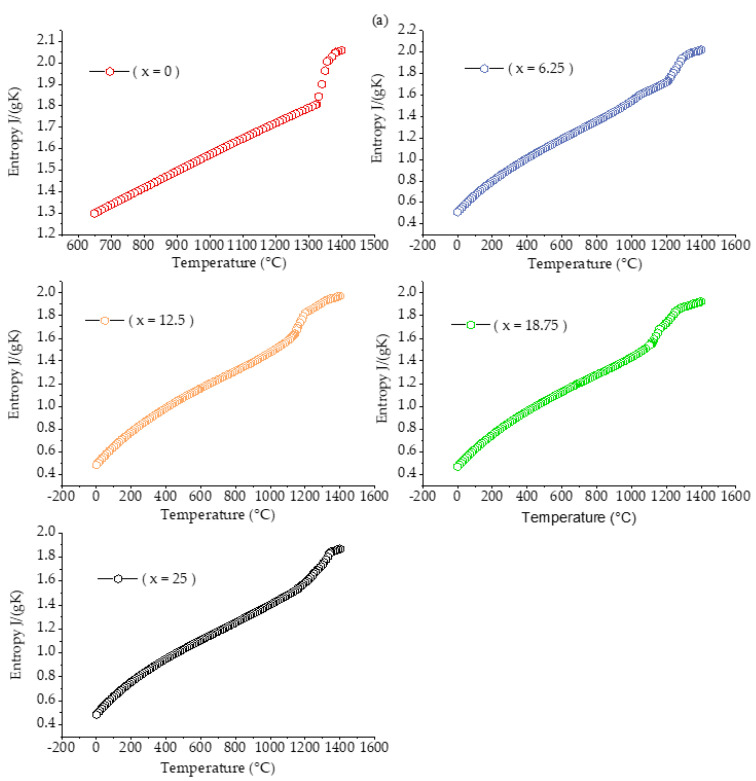

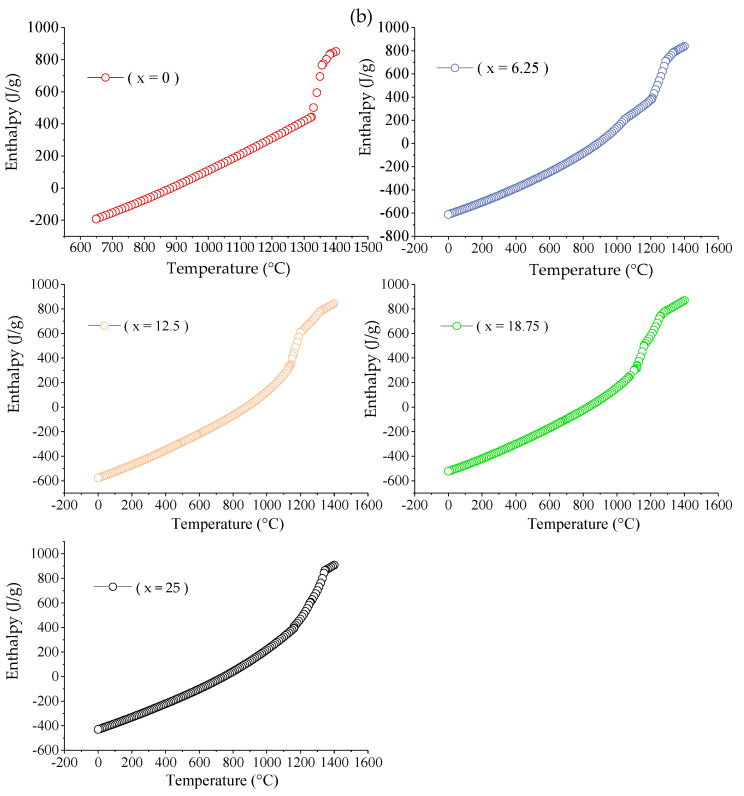
Enthalpy of mixing ΔH_mix_ (**a**) and entropy of mixing ΔS_mix_ (**b**) calculation for Fe_25_Cr_25_Ni_25_Ti_x_Al_(25−x)_ multi-principal element alloys.

**Figure 8:**
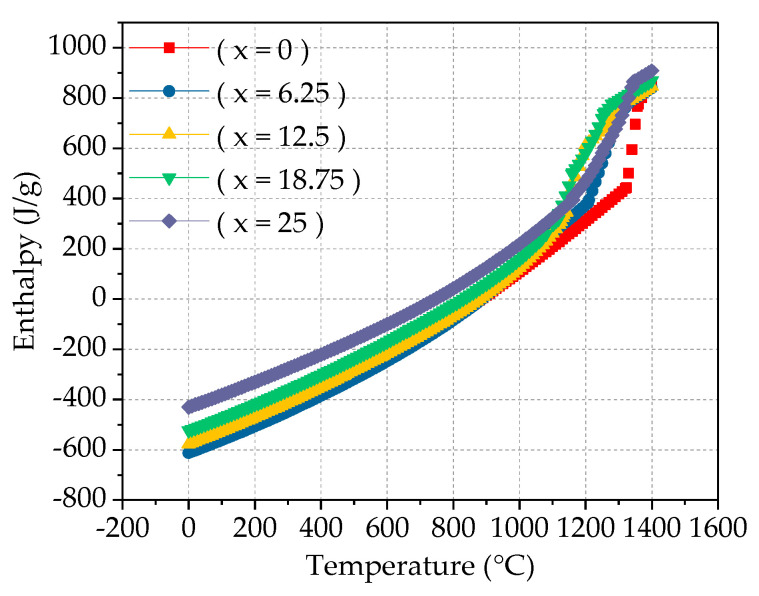
Enthalpy of mixing ΔH_mix_ calculation for Fe_25_Cr_25_Ni_25_Ti_x_Al_(25−x)_ multi-principal element alloys.

**Figure 9:**
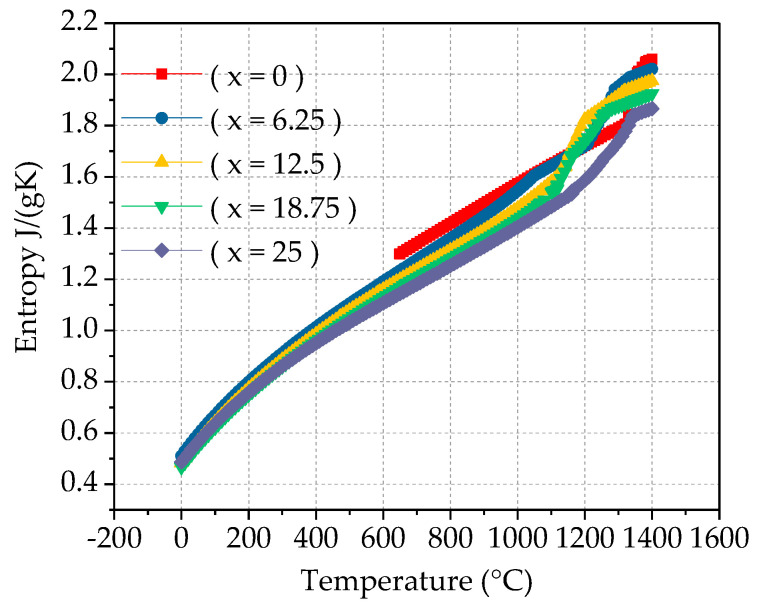
Entropy of mixing ΔS_mix_ calculation for Fe_25_Cr_25_Ni_25_Ti_x_Al_(25−x)_ multi-principal element alloys.

Enthalpy of mixing, also known as the heat of mixing, is one of the mixing functions, which refers to the linear relationship between the alloy’s transition temperature and the absolute value of the alloy’s mixing enthalpy. The mixing entropy and the mixing enthalpy are in a competitive position. Regardless of whether the mixing enthalpy is positive or negative, the increase of the mixing entropy will inevitably lead to a decrease in the system’s free energy, thereby improving the stability of the system. This article uses JMatPro software to predict the mixing enthalpy and mixing entropy of Fe_25_Cr_25_Ni_25_Ti_x_Al_(25−x)_ multi-principal element alloys, as shown in Figure 7. It can be observed from the figure that as the temperature increases, the mixing entropy and mixing enthalpy of the alloy increase, indicating that the system is more stable. The greater the absolute value of the mixing enthalpy, the higher the alloy transition temperature. The absolute value of the mixing enthalpy reflects the excess entropy value at the transition point of the alloy. By adding alloy elements or adjusting the alloy composition based on of known alloys, beneficial alloy transition temperature values can be obtained. It can be observed from Figure 8 that as the Ti content increases, the mixing entropy of the alloy gradually increases, but the overall stability tends to be stable. The mixing enthalpy gradually decreases and tends to stabilize, as shown in Figure 9.

**Figure 10:**
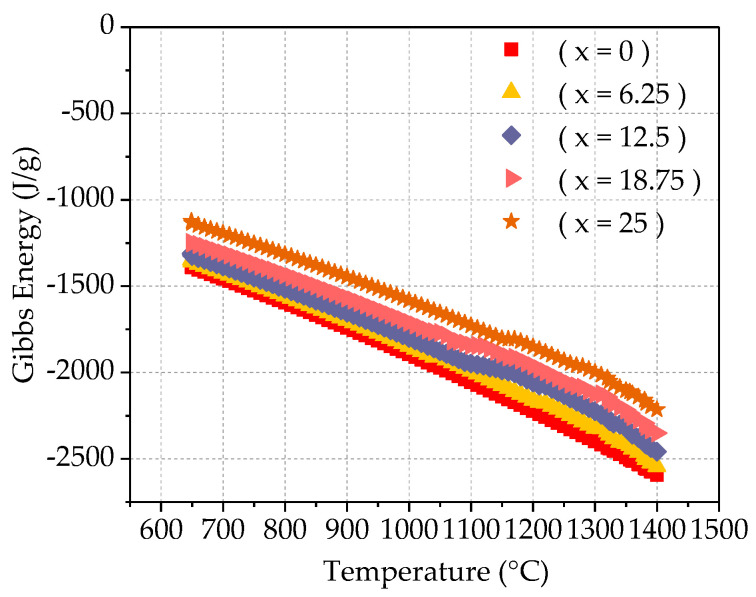
Gibbs free Energy calculation for Fe_25_Cr_25_Ni_25_Ti_x_Al_(25−x)_ multi-principal element alloys.

Gibbs free energy, also known as the Gibbs function, also known as free enthalpy, is an important thermodynamics parameter. It is often represented by G, and its definition is G = H − TS, Where H is enthalpy, T is temperature (absolute temperature, K), and S is entropy. Define ΔG = ΔH − TΔS (KJ/mol), G is called Gibbs free energy. Because H, T, and S are all state functions, G is the state function. The amount of Gibbs free energy change -ΔG = −(G2 − G1) ≥ W is not. It shows that the state function G is the ability of the system to do non-volume work under isothermal pressure and pressure. The reduction of G during the reaction-ΔG is the maximum non-volume work done by the system. This maximum is achieved in a reversible way. ΔG can judge the direction and mode of the reaction: −ΔG > W non-reaction proceeds spontaneously in an irreversible manner; −ΔG = W non-reaction proceeds in a reversible manner; −ΔG < W is not possible; if the reaction is carried out under isothermal pressure and pressure, no non-volume work is done, that is, W is not = 0 then ΔG < 0 proceed spontaneously; ΔG = 0 cannot proceed; ΔG > 0, the reverse reaction proceeds spontaneously. It can be seen that the direction in which the Gibbs free energy of the system decreases under isothermal pressure and pressure is the direction in which the chemical reaction that does not do non-volume work proceeds. The Gibbs free energy of any spontaneous process that does not do non-volume work under isothermal pressure and pressure will decrease. This paper uses JMatPro software to calculate the Gibbs free energy of Fe_25_Cr_25_Ni_25_Ti_x_Al_(25−x)_ (x = 0; 6.25; 12.5; 18.75; 25) multi-principal element alloy, as shown in Figure 10. It can be seen from the figure that as the temperature rises high, the Gibbs free energy of the system gradually decreases, indicating that the reaction proceeds spontaneously. In the isothermal and isotactic state, as the Ti content increases, the Gibbs free energy decreases and the reaction proceeds spontaneously.

### 3.4. First-Principles Prediction of Alloy Stability

**Figure 11:**
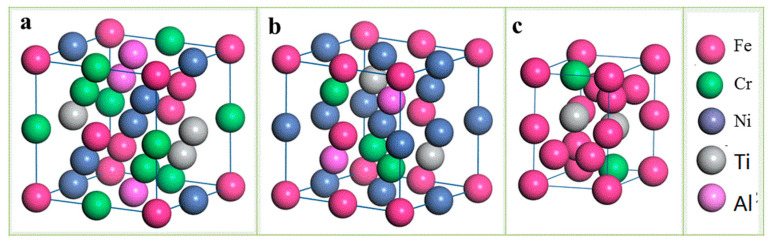
The supercells of the disordered BCC A2 structure (**a**), the ordered BCC B2 structure (**b**), and the Laves phase structure (**c**) employed in the first-principles calculations for Fe_25_Cr_25_Ni_25_Ti_12.5_Al_12.5_ multi-principle element alloys.

To quantitatively evaluate the stability and elastic properties of the phase structure of Fe_25_Cr_25_Ni_25_TixAl_(25−x)_ multi-principal element alloys, the first-principle used to calculate the formation heat, bonding energy, bulk modulus, shear modulus, and Youngs’ modulus of the alloy. The modulus, Poisson’s ratio, and the nature of the anisotropy predicted. According to the phase diagram analysis described above, the composition phase of the Fe_25_Cr_25_Ni_25_Ti_x_Al_(25−x)_ multi-principal element alloys includes a disordered BCC1 phase, an ordered BCC2 phase, and a Laves phase. The crystal structure model of the body-centered cubic structure and the close-packed hexagonal structure is established based on the Fe supercell and the Ti supercell. The Fe crystal space group of the body-centered cubic structure is IM-3M, the lattice constant is a = b = c = 2.866 Å, and the unit cell contains two atoms, and the positions are (0 0 0) and (0.5 0.5 0.5), respectively. The disordered BCC A2 phase and the ordered BCC B2 phase established on this basis contain 16 atoms. The Ti crystal space group of the close-packed hexagonal structure is P63/mmc, and the lattice constant is a = b = 2.9506 Å, c = 4.6788 Å, α = β = 90°, γ = 120°. The atomic positions in the unit cell are (0.333, 0.667, 0.25), (0.667, 0.333, 0.75), and the established Laves phase supercell contains 12 atoms. Multi-principal element alloys have higher entropy values and greater chaos, and the parent phase is disordered. Common modeling methods include Coherent Potential Approximation (CPA) [24] and Virtual Crystal Approximation (VCA) [25]. Methods such as Special Quasirandom Structure (SQS), the modeling methods in this paper are all modeled by SQS [26]. For the Fe_25_Cr_25_Ni_25_Ti_12.5_Al_12.5_ alloy studied, a 2 × 2 × 2 supercell structure containing 16 atoms was used. In the modeling stage, an alloy of a certain composition was first established, and based on this, the atomic ratio adjusted to form an alloy of different compositions. For example, a Fe_25_Cr_25_Ni_25_Ti_12.5_Al_12.5_ multi-principal element alloys was first established to establish a disordered BCC A2 phase, an ordered BCC B2 phase, and a Laves phase, respectively. Figure 11 shows the supercell structure of the disordered BCC A2 phase, the ordered BCC B2 phase, and the fully chemically disordered Laves phase in the Fe_25_Cr_25_Ni_25_Ti_12.5_Al_12.5_ alloy. Table 2 shows the lattice parameters of the supercells calculated by the direct optimization method for the Fe_25_Cr_25_Ni_25_Ti_12.5_Al_12.5_ alloy. The theoretical calculations shown in the table are in good agreement with the experimental results.

The strength and structural stability of crystals are closely related to their formation of heat and bonding energy. Heat is formed to determine which configuration is easier to form, and bonding energy is used to determine which configuration is more stable. The formation heat is defined as the difference between the energy of the compound and the corresponding composition. Usually, a negative value indicates that the substance can be stably present, and a positive value indicates that the substance is difficult to be formed. A smaller value indicates that the alloying ability is stronger. For Fe_25_Cr_25_Ni_25_Ti_x_Al_(25−x)_ (x = 0; 6.25; 12.5; 18.75; 25) system alloys, the expression of heat is:

$${\mathrm{Fe}}_{25}+{\mathrm{Cr}}_{25}+{\mathrm{Ni}}_{25}+{\mathrm{Ti}}_{x}+{\mathrm{Al}}_{\left(25-x\right)}\to {\mathrm{Fe}}_{25}{\mathrm{Cr}}_{25}{\mathrm{Ni}}_{25}{\mathrm{Ti}}_{x}{\mathrm{Al}}_{\left(25-x\right)}$$

(4) $$H_{\mathrm{form}}=\left(E_{\mathrm{total}}^{{\mathrm{Fe}}_{25}{\mathrm{Cr}}_{25}Ni_{25}{\mathrm{Ti}}_{x}{\mathrm{Al}}_{\left(25-x\right)}}-\frac{25}{100}E_{\mathrm{solid}}^{\mathrm{Fe}}-\frac{25}{100}E_{\mathrm{solid}}^{\mathrm{Cr}}-\frac{25}{100}E_{\mathrm{solid}}^{\mathrm{Ni}}\;-\frac{x}{100}E_{\mathrm{solid}}^{\mathrm{Ti}}-\frac{25-x}{100}E_{\mathrm{solid}}^{\mathrm{Al}}\right)\;$$

$E_{\mathrm{tot}}$ is the total energy of the unit cell, $E_{\mathrm{solid}}^{\mathrm{Fe}}$, $E_{\mathrm{solid}}^{\mathrm{Cr}}$, $E_{\mathrm{solid}}^{\mathrm{Ni}}$, $E_{\mathrm{solid}}^{\mathrm{Ti}}$ and $E_{\mathrm{solid}}^{\mathrm{Al}}$ respectively represent the heat of formation of a single atom in the solid element.

The bonding energy of a crystal is defined as the energy released by the combination of free atoms into crystals, or the energy required to decompose a crystal into a single atom, usually a negative value. Therefore, the smaller the value of the bonding energy, the more stable the crystal formed. For the Fe_25_Cr_25_Ni_25_Ti_x_Al_(25−x)_ alloy, the bonding energy is calculated as follows:

(5) $$E_{\mathrm{coh}}=\left(E_{\mathrm{total}}^{{\mathrm{Fe}}_{25}{\mathrm{Cr}}_{25}{\mathrm{Ni}}_{25}{\mathrm{Ti}}_{x}{\mathrm{Al}}_{\left(25-x\right)}}-\frac{25}{100}E_{\mathrm{atom}}^{\mathrm{Fe}}-\frac{25}{100}E_{\mathrm{atom}}^{\mathrm{Cr}}-\frac{25}{100}E_{\mathrm{atom}}^{\mathrm{Ni}}-\frac{x}{100}E_{\mathrm{atom}}^{\mathrm{Ti}}-\frac{25-x}{100}E_{\mathrm{atom}}^{\mathrm{Al}}\right)$$

$E_{\mathrm{atom}}^{\mathrm{Fe}}$, $E_{\mathrm{atom}}^{\mathrm{Cr}}$, $E_{\mathrm{atom}}^{\mathrm{Ni}}$, $E_{\mathrm{atom}}^{\mathrm{Ti}}$, $E_{\mathrm{atom}}^{\mathrm{Al}}$ are isolated atomic energies of a single atom, respectively.

**Figure 12:**
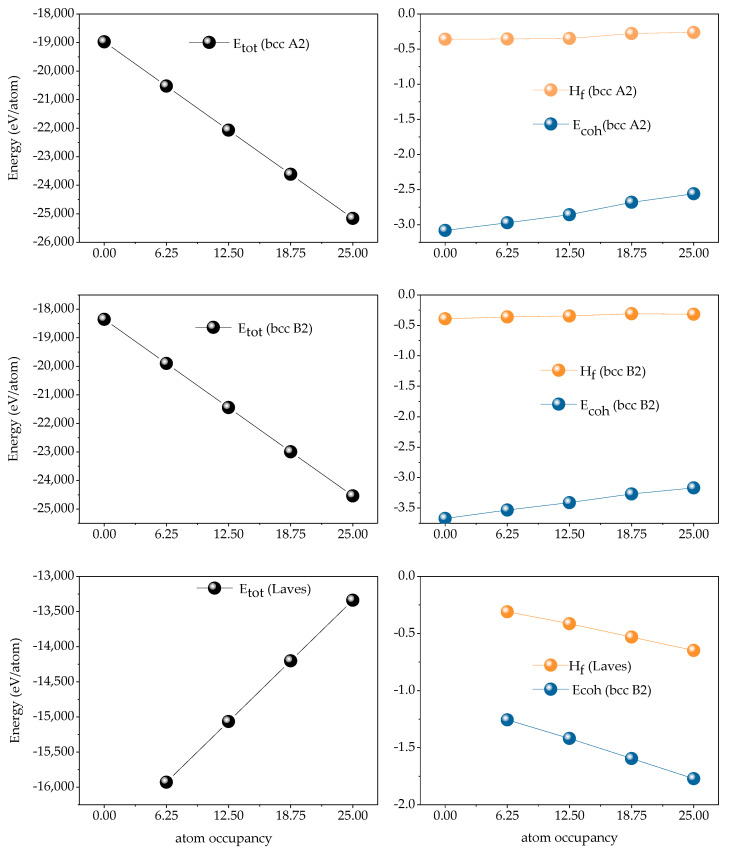
The total energy E_tot_, forming heat H_f_ and bonding energy Ec of the Fe_25_Cr_25_Ni_25_Ti_x_Al_(25−x)_ system multi-principal element alloys.

Figure 12 is the calculated total ground state energy of Fe_25_Cr_25_Ni_25_Ti_x_Al_(25-x)_ alloy atom assembly, forming heat and binding energy, and the result is negative, indicating that the alloy of the system is usually easy to form. From the energy point of view, the system of the alloy is stable. Further analysis, x = 0; 6.25; 12.5; 18.75; 25. With the increase of Ti content, the calculated total energy of bcc A2 phase and bcc B2 phase is getting smaller and smaller, indicating that the formation of the system is relatively stable. However, the calculated total energy for forming the Laves phase is getting larger and larger, indicating that the more difficult the Laves phase is to form, the system is not easy to stabilize. The formation heat of the bcc A2 phase gradually decreases and tends to be stable, and both are less than 0, indicating that it is easier to form. The binding energy gradually increases and tends to be stable and all are less than 0, indicating that the configuration with less Ti content is more stable. With the increase of Ti content, the formation heat and binding energy of Laves phase gradually decrease, indicating that Laves phase is easy to form in alloys with high Ti content and the configuration is more stable.

### 3.5. First-Principles Prediction of Alloy Elastic Properties

The elastic constant is the stiffness that characterizes the response of the crystal to the external strain ε. When a small strain is applied, the internal energy of the system has a quadratic linear relationship with the magnitude of the strain (Hooke’s law), and the elastic constant is the coefficient of the quadratic term. The expression is:

(6) $$U=\frac{\Delta E}{V_{0}}=\frac{1}{2}\overset{6}{\underset{i}{\sum }}\overset{6}{\underset{j}{\sum }}C_{ij}e_{i}e_{j}$$

The strain tensor ε is defined as:

(7) $$\varepsilon \;=\left(\begin{array}{ccc} e_{1} & \frac{1}{2}e_{6} & \frac{1}{2}e_{5}\\ \frac{1}{2}e_{6} & e_{2} & \frac{1}{2}e_{4}\\ \frac{1}{2}e_{5} & \frac{1}{2}e_{4} & e_{3} \end{array}\right),\;\mathrm{Voigt}\;\mathrm{is}\;\mathrm{marked}\;\mathrm{as}\;\left\{\begin{array}{c} \mathrm{xx}\to 1,\mathrm{yy}\to 2,\mathrm{zz}\to 3\\ \mathrm{yz}\to 4,\mathrm{xz}\to 5,\mathrm{xy}\to 6 \end{array}\right\}$$

where ΔE represents the energy difference before and after deformation of the unit cell, $V_{0}$ is the volume of the unit cell, $C_{ij}$ is the component of the elastic tensor, and $e_{i}$ and $e_{j}$ are the applied minor strains.

For a stable structure, the Born mechanical stability standard for orthorhombic crystals at zero pressure is [27]:

$$C_{11}>0,{\;C}_{22}>0,{\;C}_{33}>0,{\;C}_{44}>0,{\;C}_{55}>0,{\;C}_{66}>0,$$

$$(C_{11}+C_{33}-2C_{13})>0,\;(C_{22}+C_{33}-2C_{23})>0,$$

$$\left(C_{11}+C_{22}-2C_{12}\right)>0,$$

(8) $$(C_{11}+C_{22}+C_{33}+C_{12}+C_{13}+C_{23})>0,$$

According to the calculated single-crystal elastic constant $C_{ij}$, the polycrystalline elastic properties of the material such as the bulk modulus B, the shear modulus G, the elastic modulus E, and the Poisson can be obtained by the Voigt–Reuss–Hill (VRH) approximation method [28]. The ratios ν, Voigt, and Reuss approximate the maximum and minimum limits of polycrystalline elastic properties. The expressions are:

(9) $$B=\frac{1}{2}\left(B_{V}+B_{R}\right),\;G=\frac{1}{2}\left(G_{V}+G_{R}\right),E=\frac{9\mathrm{BG}}{3B+G}\;,\upsilon =\frac{3B-E}{6B}$$

$$B_{V}=\frac{1}{9}(C_{11}+C_{22}+C_{33})+\frac{2}{9}\left(C_{12}+C_{13}+C_{23}\right)$$

$$G_{V}=\frac{1}{15}(C_{11}+C_{22}+C_{33}+C_{12}+C_{13}+C_{23})+\frac{1}{5}\left(C_{12}+C_{13}+C_{23}\right)$$

$$B_{R}=\frac{1}{(S_{11}+S_{22}+S_{33})+2\left(S_{12}+S_{13}+S_{23}\right)}$$

(10) $$G_{R}=\frac{1}{4(S_{11}+S_{22}+S_{33})-4\left(S_{12}+S_{13}+S_{23}\right)+3\left(S_{44}+S_{55}+S_{66}\right)}$$

where *S_ij_* is the elastic compliance of the material single crystal and *S_ij_* is the inverse matrix of *C_ij_*. The V and R in the subscript represent the calculation results of the Voigt and Reuss approximation models, respectively.

For a hexagonal crystal, the standard for mechanical stability is [29]:

(11) $$C_{11}>0,{\;C}_{11}-C_{12}>0,{\;C}_{44}>0,\;\left(C_{11}+C_{12}\right)C_{33}-2C_{13}^{2}>0.$$

From the calculated single-crystal elastic constants, the polycrystalline elastic property can be approximated by Voigt–Reuss–Hill (VRH), and the expression is [30]:

(12) $$B=\frac{1}{2}\left(B_{V}+B_{R}\right),\;G=\frac{1}{2}\left(G_{V}+G_{R}\right),E=\frac{9\mathrm{BG}}{3B+G}\;,\upsilon =\frac{3B-2G}{2\left(3B+G\right)}$$

$$B_{V}=\frac{2}{9}(C_{11}+C_{12}+\frac{1}{2}C_{33}+2C_{13})$$

$$G_{V}=\frac{1}{30}(7C_{11}-5C_{12}+12C_{44}+2C_{33}-4C_{13})$$

$$B_{R}=\frac{(C_{11}+C_{12})C_{33}-2C_{13}^{2}}{C_{11}+C_{12}+2C_{33}-4C_{13}}$$

$$G_{R}=\frac{5}{2}\left\{\frac{\left[(C_{11}+C_{12}\right)C_{33}-2C_{13}^{2}]C_{44}C_{66}}{3B_{V}C_{44}C_{66}+\left[(C_{11}+C_{12}\right)C_{33}-2C_{13}^{2}](C_{44}+C_{66})}\right\}$$

**Figure 13:**
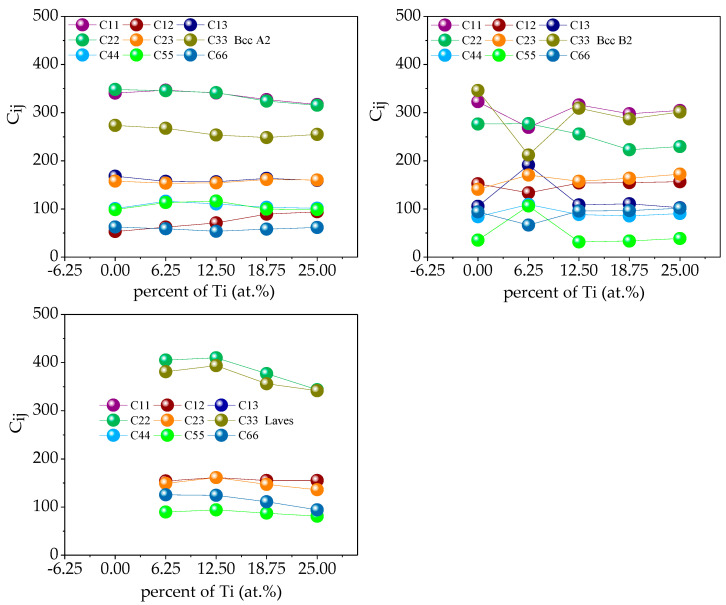
The calculated elastic constants (*C_ij_*) for single-phase Fe_25_Cr_25_Ni_25_Ti_x_Al_(25−x)_.

**Figure 14:**
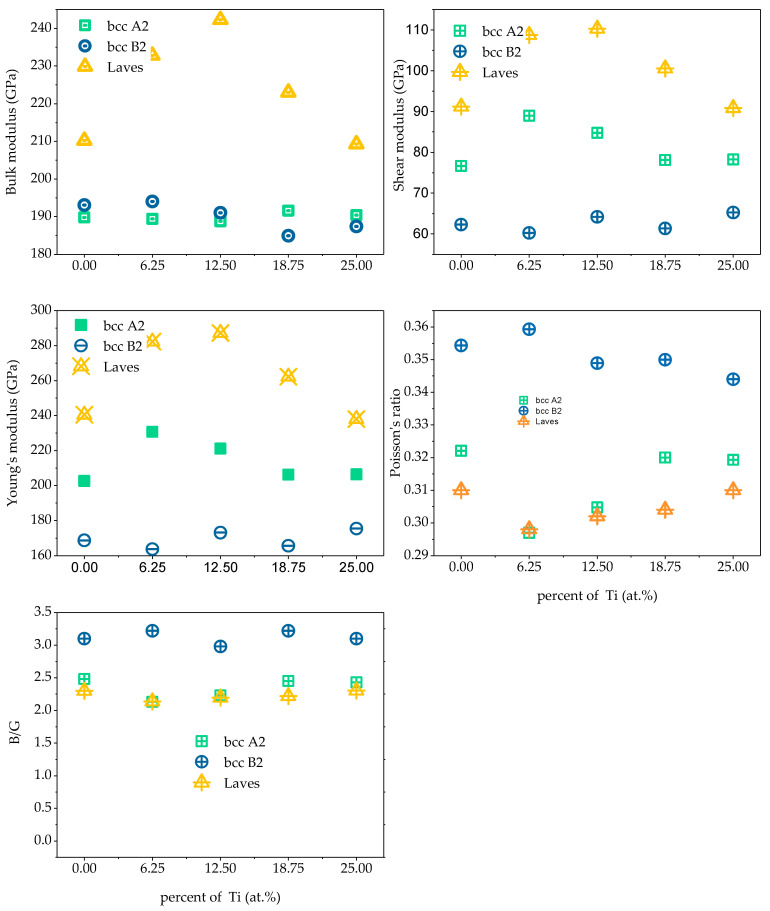
The calculated average properties (bulk modulus (B), shear modulus (G), elastic modulus (E), and Poisson’s ratio (ν)) for single-phase Fe_25_Cr_25_Ni_25_Ti_x_Al_(25−x)_ of different atom occupancy positions by using Voigt–Reuss–Hill approximation.

In the Fe_25_Cr_25_Ni_25_Ti_x_Al_(25-x)_ system multi-principal element alloys, the single-crystal elastic constants C_ij_ of the alloy calculated by the above formula are shown in Figure 13. Through verification, the single-crystal elastic constants of the five alloys all meet the mechanical stability criterion, indicating that the five multi-principal element alloys structures established are mechanically stable. According to the calculated theoretical elastic constant of the single alloy crystal, the elastic properties of the alloy polycrystal obtained by the Voigt–Reuss–Hill approximation shown in Figure 12. The bulk modulus of a crystal is the resistance of a material to uniform compression of the outside under an elastic system. Shear modulus is the ratio of shear stress to strain, which characterizes the material’s ability to resist shear strain. Young’s modulus is one of the most essential and characteristic mechanical properties of elastic materials, reflecting the softness of materials to some extent hard. Poisson’s ratio ν is commonly used to characterize the ability of a material to resist shear stress. The larger the Poisson’s ratio, the better the shape of the material. In addition to Poisson’s ratio, the ductile and brittleness of multi-principal element alloys can also be determined according to the Pugh empirical criteria [31], specifically defined as the ratio of bulk modulus to shear modulus (B/G). When B/G < 1.75, the material exhibits brittleness; when B/G > 1.75, the material exhibits toughness, and the larger the ratio, the stronger the toughness and the better the ductility of the material. Figure 14 shows the polycrystalline elastic properties of the five alloys. The values of shear modulus G and Young’s modulus E in the alloy are found to be BCC B2 phase < BCC A2 phase < Laves phase; illustrated in the Fe_25_Cr_25_Ni_25_Ti_x_Al_(25−x)_ system. Among the alloys, the Laves phase is the hardest and has the strongest resistance to shear deformation, followed by the BCC A2 phase, followed by the BCC B2 phase. When the value of bulk modulus B is x < 0.5, BCC B2 phase < BCC A2 phase < Laves phase; when x ≥ 0.5, BCC A2 phase < BCC B2 phase < Laves phase, indicating that Laves phase has the strongest volume deformation Resistance. By analyzing the Poisson’s ratio and the value of B/G, it is found that the BCC B2 phase > BCC A2 phase > Laves phase indicates that the BCC B2 phase has excellent ductility, followed by the BCC A2 phase, and the Laves phase has the worst ductility.

## 4. Conclusions

In this paper, a new type of Fe_25_Cr_25_Ni_25_Ti_x_Al_(25−x)_ multi-principal element alloys is designed by using the research idea of material genetic engineering, which can improve the ductility of the alloy. It is realized by a combination of first-principles calculation and thermodynamic analysis. The main conclusions are as follows:

(1) The phase composition, content and main constituent elements of Fe_25_Cr_25_Ni_25_Ti_x_Al_(25−x)_ (x = 0; 6.25; 12.5; 18.75; 25) alloys were successfully predicted by thermodynamic phase diagram analysis. When x = 0, the alloy consists of a disordered BCC A2 phase and an ordered BCC B2 phase. When x = 6.25; 12.5; 18.75; 25, the phase consists of a disordered BCC A2 phase, an ordered BCC B2 phase, and a Laves phase. As the content of the Ti element continues to increase, the content of the disordered BCC A2 phase and ordered BCC B2 phase in the alloy gradually decreases. The content of the Laves phase increases gradually.

(2) The structural stability and elastic properties of Fe_25_Cr_25_Ni_25_Ti_x_Al_(25−x)_ (x = 0; 6.25; 12.5; 18.75; 25) alloys were predicted by first-principles calculation based on density functional theory. The calculation results of the formation heat and bonding energy of the alloy indicate that the alloy of the system is usually formed easily, and the alloy of the system has structural stability from the viewpoint of energy. By calculating the elastic properties of the alloy, such as bulk modulus B, shear modulus G, Young’s modulus E, Poisson’s ratio ν, and B/G, the results show that the disordered BCC A2 phase and the ordered BCC B2 phase are the ductile phases, the Laves phase is brittle. The ordered BCC B2 phase has the best ductility.

(3) We predicted the mixing entropy, mixing enthalpy and Gibbs free energy of Fe_25_Cr_25_Ni_25_Ti_x_Al_(25−x)_ multi-principal element alloys. The results show that as the temperature increases, the mixing entropy and mixing enthalpy of the system are equally increase, indicating that the system tends to stabilize. With the increase of Ti content, the mixing entropy of the alloy gradually increases; the mixing enthalpy gradually decreases, but the overall stability tends to be stable. By calculating the Gibbs free energy of the system, the results show that as the temperature increases, the Gibbs free energy of the system gradually decreases, indicating that the reaction proceeds spontaneously. In the isothermal and isostatic state, as the Ti content increases, the Gibbs free energy decreases and the reaction proceeds spontaneously.

## Figures and Tables

**Table 1:** The molar percentage of each phase of the Fe_25_Cr_25_Ni_25_Ti_x_Al_(25-x)_ alloy at 800 °C.

Alloys	Mole Percent of Phase		
	BCC A2	BCC B2	Laves
Fe_25_Cr_25_Ni_25_Al_25_	53.36%	46.64%	0
Fe_25_Cr_25_Ni_25_Ti_6.25_Al_18.75_	35.19%	44.08%	20.73%
Fe_25_Cr_25_Ni_25_Ti_12.5_Al_12.5_	28.43%	34.27%	37.3%
Fe_25_Cr_25_Ni_25_Ti_18.75_Al_6.25_	25.42%	25.42%	49.13%
Fe_25_Cr_25_Ni_25_Ti_25_	22.09%	3.17%	74.74%

**Table 2:** Shows the lattice parameters of the supercells calculated by the direct optimization method for the Fe_25_Cr_25_Ni_25_Ti_12.5_Al_12.5_ alloy.

Fe_25_Cr_25_Ni_25_Ti_12.5_Al_12.5_	Calculation Results		Experimental Results [16]	
BCC A2	-	a = 2.866 Å	a = 2.988 Å	-
BCC B2	-	a = 2.998Å	a = 3.052 Å	-
Laves phase	a = 4.699 Å	c = 7.628 Å	a = 4.714 Å	c = 7.632 Å

## Data Availability

The data presented in this study are available on request from the corresponding author.
